# Integration of a perfusion reactor and continuous precipitation in an entirely membrane‐based process for antibody capture

**DOI:** 10.1002/elsc.202300219

**Published:** 2023-09-07

**Authors:** Gabriele Recanati, Magdalena Pappenreiter, Christoph Gstoettner, Patrick Scheidl, Elena Domínguez Vega, Bernhard Sissolak, Alois Jungbauer

**Affiliations:** ^1^ Department of Biotechnology University of Natural Resources and Life Sciences Vienna Austria; ^2^ Innovation Management Bilfinger Life Science GmbH Salzburg Austria; ^3^ Center for Proteomics and Metabolomics Leiden University Medical Center Leiden The Netherlands

**Keywords:** cross flow filtration, IgG, integrated processing, precipitation

## Abstract

Continuous precipitation coupled with continuous tangential flow filtration is a cost‐effective alternative for the capture of recombinant antibodies from crude cell culture supernatant. The removal of surge tanks between unit operations, by the adoption of tubular reactors, maintains a continuous harvest and mass flow of product with the advantage of a narrow residence time distribution (RTD). We developed a continuous process implementing two orthogonal precipitation methods, CaCl_2_ precipitation for removal of host‐cell DNA and polyethylene glycol (PEG) for capturing the recombinant antibody, with no influence on the glycosylation profile. Our lab‐scale prototype consisting of two tubular reactors and two stages of tangential flow microfiltration was continuously operated for up to 8 days in a truly continuous fashion and without any product flow interruption, both as a stand‐alone capture and as an integrated perfusion‐capture. Furthermore, we explored the use of a negatively charged membrane adsorber for flow‐through anion exchange as first polishing step. We obtained a product recovery of approximately 80% and constant product quality, with more than two logarithmic reduction values (LRVs) for both host‐cell proteins and host‐cell DNA by the combination of the precipitation‐based capture and the first polishing step.

**Abbreviations**
AEX: anion exchange
CCCS: clarified cell culture supernatant
CHO: Chinese hamster ovary cells
hcDNA: host‐cell DNA
HCPs: host‐cell proteins
HF: hollow fiber
ID: internal diameter
PEG: polyethylene glycol
RTD: residence time distribution
TFF: tangential flow filtration
TMP: transmembrane pressure
VCC: viable cells concentration
VVD: vessel volumes per day

## 1 INTRODUCTION

Continuous precipitation coupled with continuous tangential flow filtration offers a cost‐effective alternative for the capture of recombinant antibodies from crude cell culture supernatant. The removal of surge tanks between unit operations, by the adoption of tubular reactors, maintains a continuous harvest and mass flow of product with the advantage of a narrow residence time distribution (RTD). Despite the great purification potential of protein A affinity chromatography, even with multi‐column approaches the harvest of product happens in cycles due to the bind‐and‐elute nature of the chromatographic step. After capture, further polishing steps are typically required to reduce impurities concentration to an acceptable level and meet the FDA guidelines requirements. In most cases, the first step involves anion‐exchange chromatography (AEX). Host cell DNA (hcDNA) and large part of the host cell proteins (HCPs) possess a net negative charge at neutral pH [1], while most recombinant antibodies have instead a basic PI and are positively charged in these conditions. Therefore, product and impurities are separated based on their different charge via flow‐through AEX (FT‐AEX). For flow‐through technologies, parallelization (e.g., twin FT‐AEX columns) allows for an uninterrupted harvest of product at the outlet of the unit operation. In this regard, more effort was carried on in recent years to develop entirely flow‐through polishing of recombinant antibodies with the aim of generating a truly continuous mass flow of product and reducing process complexity [2, 3, 4, 5, 6].

The use of precipitation and filtration techniques for the capture and harvest of recombinant antibodies from clarified cell culture supernatant (CCCS) was also intensively explored over the last decade [7, 8, 9, 10, 11, 12, 13, 14, 15, 16, 17, 18, 19]. PEG precipitation principles are well understood [7, 8, 20, 21, 22], however, integrated processes have not yet been established. Precipitation is a cost‐effective alternative for the capture of recombinant antibodies from CCCS and continuous operation is achieved in tubular reactors equipped with static mixers [23, 24]. Multiple precipitation steps can be performed in a process, targeting product or impurities [25, 26, 27]. When the latter are precipitated, the stream can be easily clarified in continuous by tandem depth filtration and a simple automated system can be implemented for filters exchange, by monitoring the depth filter inlet pressure and outlet turbidity [28]. When the product is precipitated (e.g., an antibody), it can be concentrated and washed by multiple stages of TFF. The adoption of TFF for the harvest of precipitate results in high redissolution yield after concentration and diafiltration, due to the lower compaction of the precipitate in comparison with centrifugation [13, 15]. TFF is implemented in continuous either in single‐pass or feed‐and‐bleed configuration. For the latter, part of the retentate is drawn off (also called “bleed”) to the next stage and the remaining part is recirculated. In this way, higher conversions and higher shear rates (s^−1^) are obtained across the module, with enhanced impurities removal and lower and slower membrane fouling [18].

**PRACTICAL APPLICATION**

In this work, we proved the feasibility of precipitation for the continuous capture of monoclonal antibodies. The integration of upstream and downstream in a pool‐less fashion reduces plant footprint and capital investments, while narrowing the RTD of the entire process train. Process robustness was confirmed by comparison of the downstream process performance as a stand‐alone device and integrated with a perfusion process. The scale‐up is simplified, as the whole downstream train is completely membrane‐based.

Compared to continuous chromatography, continuous precipitation coupled with continuous TFF has the advantage that the mass flow of product is never interrupted and remains fully continuous. Moreover, the absence of bind‐and‐elute steps facilitates the direct integration of unit operations in a pool‐less fashion. The removal of surge tanks reduces plant footprint and capital investments while narrowing the RTD. A narrow RTD enables process fast start‐up and shut‐down, partly simplifying the dilemma of batch definition in continuous manufacturing.

In this work, we developed continuous precipitation utilizing combined precipitation methods, CaCl_2_ for the precipitation of hcDNA (and partly HCPs) and PEG_6000_ for the capture of the recombinant antibody. We screened depth filters for the removal of the precipitated hcDNA after the first precipitation stage and evaluated the critical flux for different hollow fiber (HF) modules geometries, to select the best for our purpose. We ultimately connected all unit operations in a pool‐less fashion. The flow at the outlet of one unit was directly fed at the inlet of the following one with no surge tanks. Furthermore, we explored the use of membrane adsorbers for the first polishing step. Finally, we determined the RTD of our capture skid as suggested from the FDA and EMA guidelines for continuous manufacturing [29].

## 2 MATERIALS AND METHODS

### 2.1 Cell culture

In this study, a recombinant IgG1 (commercial name trastuzumab, PI 8.4‐8.6), was used. CHO cells were cultivated in perfusion mode in a Labfors 5 benchtop bioreactor (Infors Ht, Switzerland). Cell expansion was performed in shake flasks cultures (Corning, New York, US) with a working volume up to 500 mL. The bioreactors were inoculated at a cell density of 3–5 × 10^6^ cells/mL. The perfusion was initiated on the same day at 0.5 VVD by continuous supply of fresh perfusion medium and removal of harvest from the permeate. A perfusion rate of 0.5 VVD was maintained until a cell density of 10 × 10^6^ cells/mL and was increased stepwise with the cell density until 1 VVD until the setpoint for viable cell concentration (VCC) of 60 ± 10 × 10^6^ cells/mL. Cell bleeding was applied via an automated bleed feedback loop using VCC prediction from an on‐line capacitance probe (Incyte, Hamilton, Switzerland). The working volume was kept at 2 L by equal inflow and outflow rates. Feedback loops of bioreactor weight, feed and bleed were used to adjust the pump flow rates. The cultures were perfused using a medium composed of 4Cell XtraCHO Production Medium (Sartorius) with feed A and feed B (6%/1%) supplement (Sartorius) and 60 mM D‐glucose. The setpoints of process temperature, dissolved oxygen concentration and culture pH were 37°C, 40% and 7.0 ± 0.1, respectively. For the collection of supernatant for process development and batch‐capture, the harvest of supernatant from the bioreactor was performed with an ATF 2 (Repligen, Waltham, MA) cell retention device equipped with a polyether sulfone HF with a pore size of 0.2 µm and a surface area of 0.13 m^2^ at shear rate of 1500 s^−1^ (Repligen, Waltham, MA). For the integrated perfusion‐capture, the harvest of supernatant was performed via TFF with a polyvinylidene fluoride HF (Asahi Kasei, Tokyo, Japan) with a pore size of 0.2 µm and membrane area of 0.08 m^2^ at a shear rate of 1000 s^−1^. A magnetically levitating pump (Levitronix, Framingham, MA, US) was used for recirculation.

### 2.2 hcDNA precipitation by CaCl2

To define the best conditions for efficient hcDNA precipitation, increasing concentrations of CaCl_2_ from 0 to 350 mM were added to CCCS from a 1 M stock solution of CaCl_2_ in 100 mM MOPS, pH 7.0. The screening was performed in 1.5 mL centrifuge tubes with a total working volume of 1 mL. Samples were gently agitated for 5 min on the end‐over‐end shaker (Stuart rotator SB3; Cole‐ Parmer, Vernon Hills, IL). This time was sufficient for the maximal hcDNA precipitation at the corresponding CaCl_2_ concentration. After precipitation, samples were centrifuged at 6000 *g* for 6 min and the supernatant was collected in fresh centrifuge tubes for subsequent analysis.

### 2.3 Depth filtration of precipitated impurities

For the removal of CaCl_2_‐precipitated impurities from the product stream, depth filters capsules with various pore ratings and from two different manufacturers were tested. For turbidity measurements, a portable 2100Q turbidimeter was employed (HACH Lange GmbH, Düsseldorf, Germany). For inlet pressure recording, a PressureMAT equipped with a gauge pressure sensor for lab‐scale was used (PendoTech, New Jersey, USA). Qubicon online software (Qubicon, Vienna, Austria) allowed for the online monitoring and recording of the inlet pressure throughout the experiments. For each depth filter tested, 100 mM final concentration of CaCl_2_ was added to CCCS from a 1 M stock solution of CaCl_2_ in 100 mM MOPS, pH 7.0 in common laboratory glassware. The precipitate was kept in suspension with a magnetic stirrer. Depth filters were flushed with 40 L/m^2^ of deionized water prior to use. A peristaltic pump (114 DV, Watson Marlow, Guntramsdorf, Austria) was used to feed the solution to the inlet of the depth filter at 1.7 mL/min (40 LMH) up to a volumetric loading of 250 L/m^2^. Turbidity measurements were performed at the depth filter outlet every 30 min and on the pool of depth‐filtered solution. PharMed BPT hoses (Saint‐Gobain, Courbevoie, France) were used at the pump‐head and connected with Masterflex luer lock fittings to silicone hoses (Tygon R‐3603, Saint‐Gobain, Courbevoie, France) for the depth filters feed and outlet lines. All hoses had an inner diameter (ID) of 3.2 mm (3/32 in).

### 2.4 Trastuzumab precipitation by PEG6000

Increasing concentrations of PEG_6000_ in the range of 0 to 15% (w/w) were added to CCCS from a 50% stock solution of PEG_6000_ in 100 mM MOPS pH 7.0. CCCS was alternatively pre‐treated with a 100 mM of CaCl_2_ final concentration to assess the impact of CaCl_2_ precipitation on trastuzumab capture by PEG_6000_. Experiments were performed in 1.5 mL centrifuge tubes with 1 mL working volume. After addition of PEG_6000_, samples were incubated for 15 min on the end‐over‐end shaker (Stuart rotator SB3; Cole‐ Parmer, Vernon Hills, IL). For subsequent analysis, samples were centrifuged at 2000 *g* for 2 min and the supernatant was collected in fresh centrifuge tubes.

### 2.5 Critical flux experiments

Critical flux experiments were performed according to [30] on different HFs. The precipitate was fed at a concentration of 5 g/L with a peristaltic pump (313 DV Watson Marlow, Guntramsdorf, Austria) from a 100 mL reservoir to the inlet of the HF, with retentate and permeate recirculated back to the same reservoir. A second peristaltic pump (114DV, Watson Marlow, Guntramsdorf, Austria) was used to control and modulate the permeate flux, which was varied in a range of 11 LMH to 109 LMH. The transmembrane pressure (TMP) was monitored by installation of gauge pressure sensors for lab‐scale (PendoTech, New Jersey, US) at inlet, retentate and permeate lines of the HF. The gauge pressure sensors were connected to a PressureMAT (PendoTech, New Jersey, USA) and Qubicon online software was used for monitoring and recording.

### 2.6 Redissolution of trastuzumab at low pH

Redissolution of captured trastuzumab was performed batch‐wise with a 50 mM phosphate buffer pH 2.5 at a volumetric dilution sample:buffer of 1:5. Samples were incubated for 60 min at low pH (<3.5) on the end‐over‐end shaker (Stuart rotator SB3; Cole‐ Parmer, Vernon Hills, IL) and neutralized by addition of 200 mM phosphate buffer pH 8.0 with a volumetric dilution sample:buffer of 4:1. This ratio resulted in a solution with final pH 6.5. Samples were then immediately analyzed, stored at 4°C or frozen at −20°C for prolonged storage.

### 2.7 Continuous 2‐stages precipitation and 2‐stages TFF

The supernatant harvested during a perfusion process was either pooled in single‐use 10 L Flexboy 2D bags (Sartorius, where) and frozen at −20°C prior to its use for the stand‐alone capture of trastuzumab or directly fed to the first precipitation stage in a pool‐less integrated perfusion‐capture. Stock solutions of CaCl_2_ and PEG_6000_ were prepared accordingly to previous experiments. All solutions were sterile‐filtrated with 0.2 µm filters Sartopore 2 XLG capsule (Sartorius) in previously autoclaved plastic bottles equipped with 3‐way caps (Nalgene, Rochester, US). Peristaltic pumps were used to move fluids. All hoses used in the set‐up were silicone hoses (3.2 mm ID) or PharMed BPT hoses (Saint‐Gobain, Courbevoie, France, 3.2 mm ID) at the peristaltic pump heads. The tubular reactors were built in‐house by inserting static mixers (HT‐40‐6.30‐24‐AC; Material Acetal; Stamixco AG, Wollerau, Switzerland) in flexible standard lab hoses (Tygon R‐3603, 4.8‐mm inner diameter; Saint‐Gobain, Courbevoie, France), spirally arranged and vertically stacked. The tubular reactors length was determined upon the needed residence time for efficient precipitation (5 min for CaCl_2_ precipitation, 15 min for PEG_6000_ precipitation). Yield and recovery (%) were calculated by mass balance, with the latter including mAb losses due to filters exchange and in the permeates (e.g., at day 2 in the stand‐alone process and at day 3 in the integrated perfusion‐capture process, due to the failure of the pump for the addition of PEG_6000_).

### 2.8 Flow‐through anion exchange membrane chromatography

The precipitate harvested during the integrated perfusion‐capture process was dissolved at low pH as reported in Section 2.6. For process optimization, we applied a DoE approach. The software used for the design of experiments and statistical evaluation was Design‐Expert (Stat‐ease inc.). The solution pH was neutralized to the required pH by titration with 0.02 M NaOH. The conductivity was adjusted by addition of 1 M NaCl or by dilution with HQ‐water. For these experiments, an Äkta pure 25 (Cytiva) and a negatively charged membrane adsorber, the 3 M Polisher ST (1 cm^2^ area), were used. Prior to the loading, the device was flushed with 400 L/m^2^ of 50 mM phosphate buffer for which pH and conductivity were adjusted according to the feed solution. 50 mL (500 L/m^2^)of re‐dissolved trastuzumab was loaded on the device at a flow rate of 1 mL/min (600 LMH), as suggested from the manufacturer. The flow‐through was pooled and stored at 4°C before analysis.

### 2.9 Pulse injection experiments

The RTD for trastuzumab was determined by pulse injections experiments on all unit operations in which the antibody is in form of precipitate (the PEG_6000_ tubular reactor and the two stages of TFF). The precipitated antibody is retained in the retentate of the HF. To mimic the path followed by the antibody with salt, the permeate lines were kept closed during the experiments. The outlet of the system was connected to an Äkta pure 25 (Cytiva) for conductivity recording. First, the system was flushed with deionized water until the conductivity was below 0.3 mS/cm. Pulse injections with a total volume of 10 mL of 1 M NaCl were performed by switching the feed line from deionized water to the salt solution and back. We applied the tank‐in‐series model with increasing number (*N*) of continuously stirred tank reactors (CSTR) to fit our experimental data. The cumulative distribution function (*F*) for a series of ideal‐stirred tanks is given by equation 1:

(1) $$F=1-e^{-N\theta }\left[1+N\theta +\frac{{\left(N\theta \right)}^{2}}{2!}+\frac{{\left(N\theta \right)}^{N-1}}{\left(N-1\right)!}\right]$$

where θ refers to the total loading volume [mL] normalized by the system volume [mL].

### 2.10 HCPs concentration determination, Bradford assay and ELISA

For the small‐scale screening experiments carried out during process development, the Bradford assay was employed for HCPs determination as previously described in the literature [31]. HCPs concentration was determined by mass balance on CCCS and the supernatant of samples generated during the experiments, after centrifugation of the precipitated or not re‐dissolved matter at 2000 *g* for 2 min. For continuous processing, HCPs determination was performed with a 3rd generation CHO HCP ELISA kit (Cignus technologies, Southport, US).

### 2.11 hcDNA concentration determination

The concentration of double‐stranded DNA (dsDNA) in the samples generated through‐out the experiments was measured with a Quant‐iT Picogreen dsDNA assay kit (Life Technologies, Carlsbad, CA, USA) according to the manufacturer's instructions and as previously described in the literature [16].

### 2.12 Analytical and preparative protein A affinity chromatography, analytical size‐exclusion chromatography (SEC)

Analytical and preparative protein A affinity chromatography and analytical SEC were performed as described in a previous work [32].

### 2.13 Glycosylation analysis

Antibody samples were directly analyzed on an U3000 nanoRSLC system (Thermo Fisher Scientific). 5 µL of sample were injected onto a C4 trap column (5.0 × 0.3 mm i.d., Acclaim PepMap; Thermo Fisher Scientific) using a flow rate of 15 µL/min and a mobile phase consisting of H_2_O + 0.1% trifluoroacetic acid (TFA; Merck, Darmstadt), at a temperature of 60°C for 5 min. After trapping the antibodies, the 6‐port valve was switched and the trap was set in‐line with the separation column (diphenyl reversed phase column (15.0 × 0.1 mm ID, Halo Bioclass, 1000 Å pore size; Advanced Material Technology, Wilmington, DE). Separation of the mAb samples was conducted using a flow rate of 1.0 µL/min at a temperature of 80°C. A multi‐step gradient using solvent A (H_2_O (ELGA Labwater, Ede, the Netherlands) + 0.1% TFA) and solvent B (acetonitrile (Actu‐All Chemicals, Oss, the Netherlands) + 0.1% TFA) was programmed as follows: 20.0% B for 5 min, 20.0% to 32% in 1.0 min, 32%–50% B in 12 min, 50.0%–60.0% B in 2 min followed by 90.0% B for 5 min and 20% B for 5 min.

The LC system was coupled to a qTOF mass spectrometer (Maxis HD) via a nano‐ESI source (CaptiveSpray; both from Bruker, Bremen, Germany). Acetonitrile‐enriched nitrogen dopant gas was employed at a pressure of 0.40 bar (nanoBooster system; Bruker). Drying gas was applied with a flowrate of 3.0 L/min at a temperature of 220°C. The system was operated in positive ion mode with a capillary voltage of 1200 V. The in‐source CID energy was set to 180 eV, the quadrupole ion energy to 5.0 and the collision cell energy to 5.0 eV. A pre‐pulse storage time of 25.0 µs and a transfer time of 200.0 µs were used. The mass range was set from m/z 1000 ‐ 6000 including a rolling average of three scans. Data analysis was performed using the Compass DataAnalysis software (Bruker). Deconvolution of raw mass spectra was achieved by the maximum entropy algorithm using a resolution of 5000 and the data spacing to 1.0.

## 3 RESULTS AND DISCUSSION

### 3.1 Development of CaCl2 and PEG6000 precipitation steps

**FIGURE 1:**
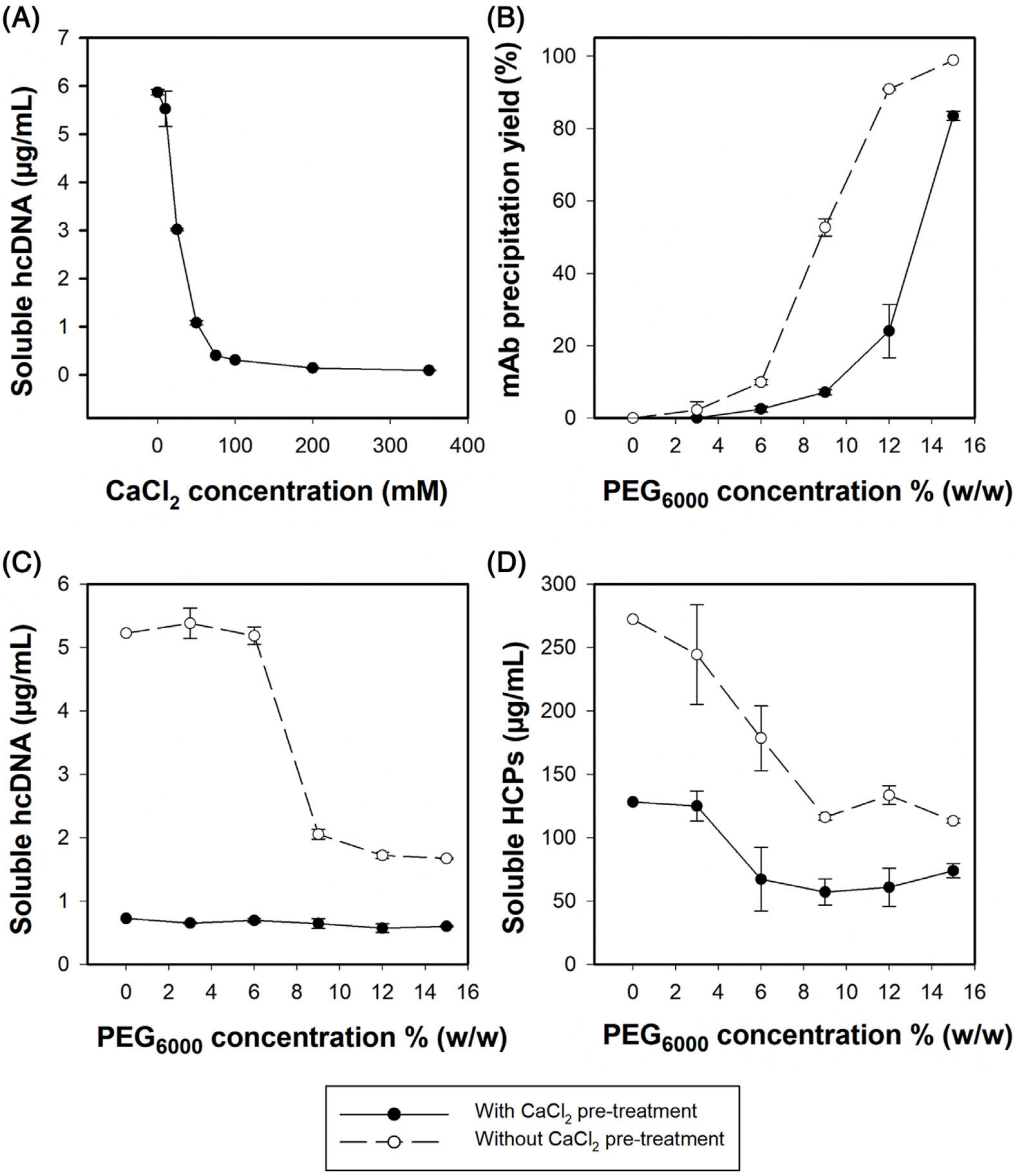
Process development for CaCl2 and PEG6000 precipitation steps. (A) Concentration of soluble hcDNA at increasing concentrations of CaCl2 (0–350 mM). (B–D) Trastuzumab precipitation yield, concentration of soluble hcDNA, concentration of soluble HCPs with (white circles, dashed line) or without (black circles, solid line) CaCl2 pre‐treatment of cell culture supernatant at increasing concentrations of PEG6000 (0–15% w/w). Error bars represent the standard deviation of triplicate determinations.

CaCl_2_ precipitation was implemented in the process to reduce soluble hcDNA content. Increasing CaCl_2_ concentrations in the range of 0 to 350 mM at pH 7.0 were added to the CCCS (Figure 1A). 100 mM CaCl_2_ was sufficient to precipitate most of the hcDNA present in CCCS and this salt concentration was used for all further experiments.

The Presence of CaCl_2_ increased the solubility of the antibody (Figure 1B). As Ca^2+^ is a chaotropic ion, it favors a salting‐in behavior according to the Hofmeister series [33]. Consequently, a higher PEG concentration is necessary to achieve the same precipitation yield, compared to the un‐treated CCCS. Addition of CaCl_2_ led to a reduction of HCPs and hcDNA of two‐fold and 22‐fold, respectively. Instead, extensive co‐precipitation of hcDNA, HCPs and trastuzumab occurred when PEG_6000_ precipitation alone was performed on CCCS (Figure 1C,D). Co‐precipitation hinders the purification of the protein of interest (POI), leading to the concomitant concentration of impurities and product during TFF. Moreover, hcDNA reduction (in our case prior to capture) improves TFF filtration performance and reduces membrane fouling over time [34]. We conclude that the addition of CaCl_2_ to CCCS prior to capture mitigates both the aforementioned issues.

### 3.2 Depth filters screening and long‐term test

**FIGURE 2:**
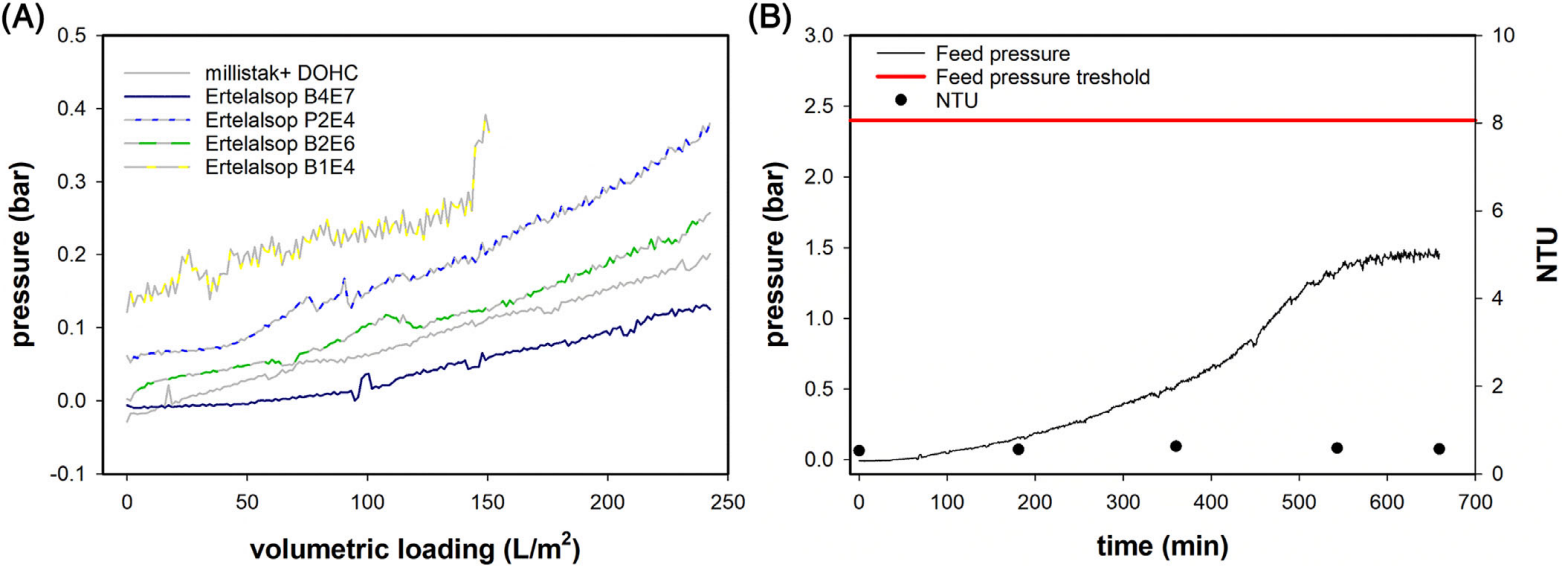
Screening and testing of depth filters for removal of precipitated hcDNA after addition of 100 mM CaCl2 final concentration to cell culture supernatant. (A) screening of depth filters over a volumetric loading of 250 L/m^2^. (B) long‐term test for the depth filter Ertelalsop B4E7 for the implementation in a continuous process. Both screening and long‐term test were performed at a feed flow rate of 1.7 mL/min (40 LMH).

Continuous depth filtration can be achieved by implementing redundancies and operating filters in a tandem mode. However, the size of the filters must fit to the mass flow of product to avoid frequent exchange of these devices, which poses a risk of process failure. We screened double layer depth filters with different media and pore size rating from two different suppliers in terms of inlet feed pressure and turbidity of the outlet clarified stream. All filters reduced the outlet turbidity below 10 NTU. Considering this, only the inlet feed pressure is reported for simplicity in Figure 2A. The depth filter Ertelalsop B4E7 produced the lowest increase in feed pressure during the screening. In a long‐term experiment, the inlet pressure of this device remained far below the pressure threshold given by the manufacturer (2.4 bar) over approximately 11 h of operation at the given feed flux of 1.7 mL/min (40 LMH), with no outlet turbidity increase (Figure 2B). This filter was selected for use in the continuous process with an expected exchange rate of two filters per day.

### 3.3 Critical flux evaluation for different hollow fibers geometry

Critical flux determination is useful for selection of HF modules for specific applications. The precipitate generated under defined conditions and concentration is recirculated on the module while the permeate flux is step‐wise increased. The critical flux is arbitrarily defined as the permeate flux above which the TMP increases sharply. For continuous manufacturing, HFs are operated far below the critical flux and usually in the range of 1 to 15 LMH. Nevertheless, critical flux determination can be used as a parameter to compare different modules and select for the best performing one in specific process conditions. A TMP rise equivalent to a maximum of 60 Pa/min is a common threshold. With this fouling rate, one HF can be operated for 24 h before being replaced [30].

**FIGURE 3:**
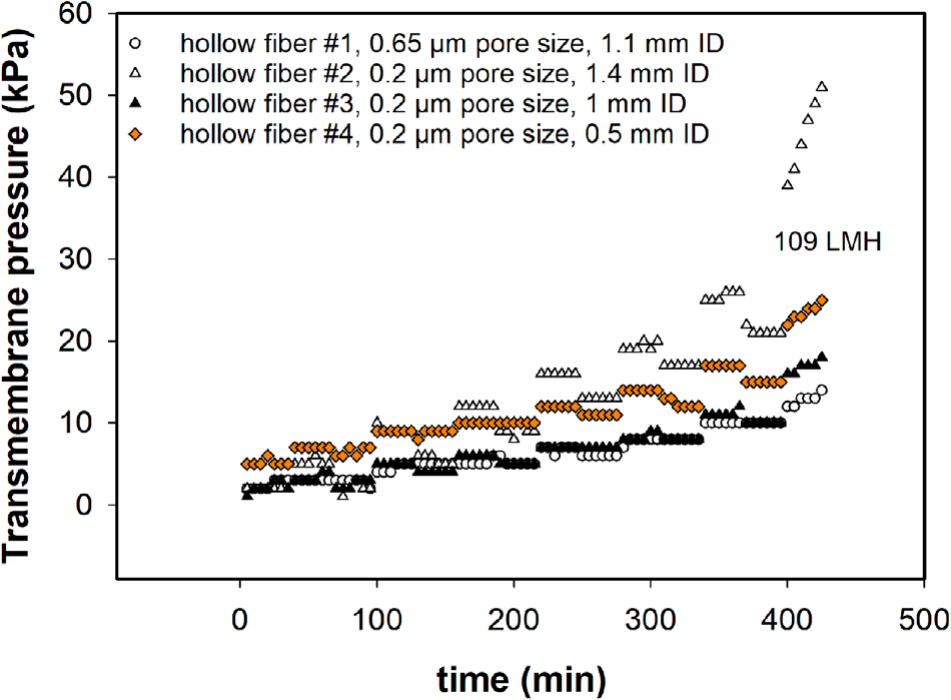
Critical flux experiments of hollow fiber modules performed in recycle mode at a shear rate of 1600 s^−1^ and precipitated monoclonal antibody concentration of 5 g/L in 18% PEG6000, 100 mM MOPS buffer, pH 7.0. Pore size and internal diameter (ID) for each module is reported in the figure legend, along with the maximum critical flux (LMH). Modules #1, #2, #3 had a path length of 30 cm. Only module #4 had a path length of 20 cm.

We screened membranes with different geometries such as fiber inner diameter, pore size and path length. All HFs were tested at a shear rate of 1600 s^−1^. Unfortunately, no correlation between modules geometry and critical flux could be determined. The critical flux for all tested modules was 109 LMH (Figure 3). Our results are in agreement with previous studies, where a critical flux of 107 LMH was observed in similar precipitation conditions and recombinant antibody concentration [35]. Ultimately, we selected HF modules according to previous works and depending on their commercial availability.

### 3.4 Continuous processing, stand‐alone capture and integrated perfusion‐capture

**FIGURE 4:**
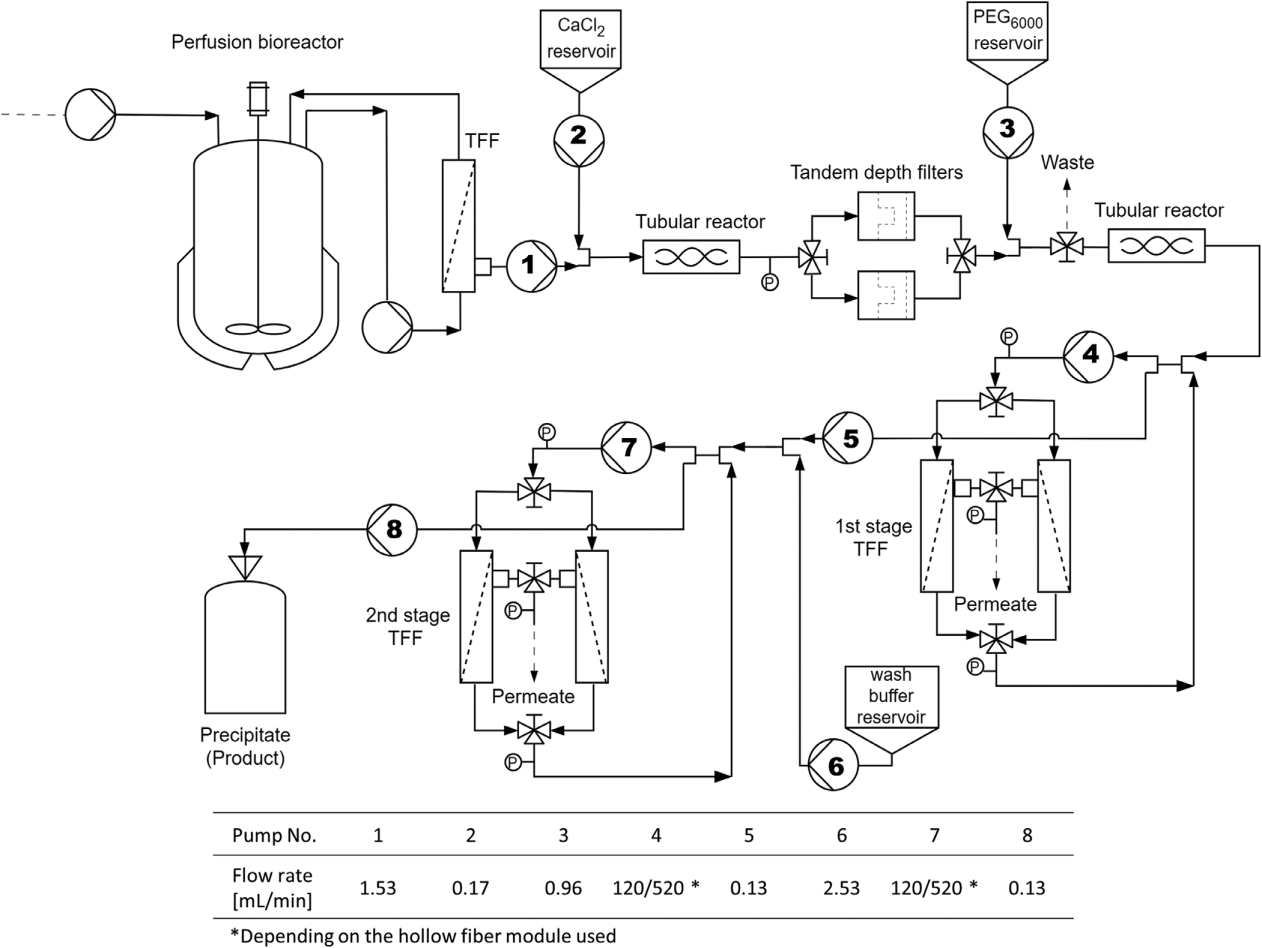
Flow diagram of the integrated perfusion‐capture set‐up. The clarified cell culture supernatant harvested from the perfusion bioreactor cell retention device (TFF) is subjected to two sequential precipitation stages (CaCl2 and PEG6000, respectively), separated from each other by a depth filtration stage. The precipitated antibody is concentrated in the first stage of TFF and bled to the second stage of TFF where it is washed by addition of fresh buffer, re‐concentrated and collected as precipitate.

**FIGURE 5:**
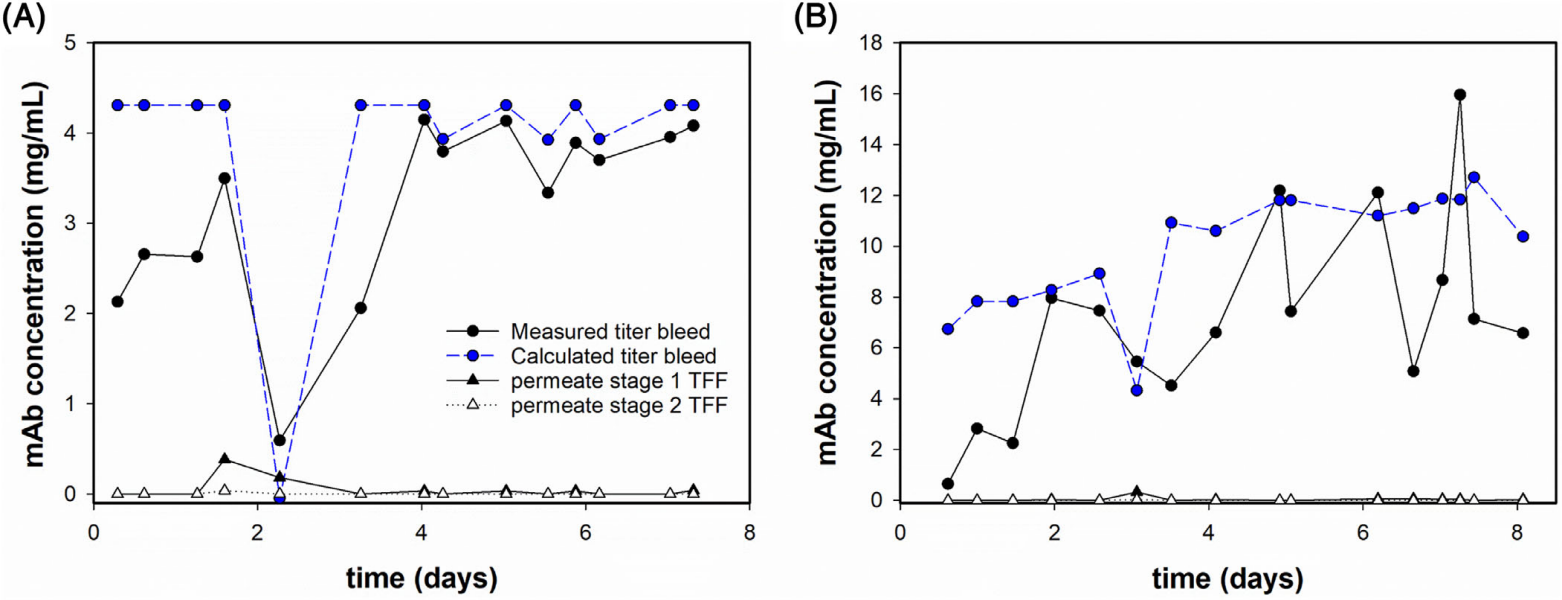
Trastuzumab concentration profile. Black circles, solid line, trastuzumab concentration profile in the bleed at the outlet of the capture unit; black triangles, solid line and white triangles, dotted line, trastuzumab concentration profile in the permeates of stage 1 and 2 of TFF, respectively; blue circles, blue dashed line, trastuzumab concentration profile at the outlet of the capture unit calculated via mass balance based on perfusion titer and losses in the permeates of TFF. (A) stand‐alone capture and (B) integrated perfusion‐capture.

A flow diagram of the entire system is shown in Figure 4. With our custom‐made set‐up, we ran a continuous process for up to 8 days either as a stand‐alone capture or as an integrated perfusion‐capture. The overall process recovery was 84.5% and 78.4 % for the stand‐alone and the integrated processes, respectively. A comparison of perfusion productivity and impurities reduction is reported in Table 1. For the stand‐alone capture, trastuzumab titer in the bleed was constant over the last 3 days of run (Figure 5A). Small oscillations of product concentration in the bleed are due to the exchange of filters and HF modules (Cytiva, 0.011 m^2^). During the integrated process, the available modules had larger membrane area (Asahi Kasei, Microza, 0.02 m^2^), thus a larger dead volume. This resulted in higher oscillations of trastuzumab concentration after HF modules exchange (Figure 5B).

**TABLE 1:** Comparison of perfusion productivity, capture step recovery and impurities reduction for the stand‐alone capture and for the integrated perfusion‐capture processes.

	Stand‐alone	Integrated
PVP g · (L · day) ^−1a^	0.4	1.0
Processed volume (L/day)	2	2
Purified mAb (g)	4.5	11.9
HCPs (LRV)	1.1	1
hcDNA (LRV)	1.7	1.9
Process recovery (%)	84.5	78.4

^a^ Perfusion volumetric productivity.

**FIGURE 6:**
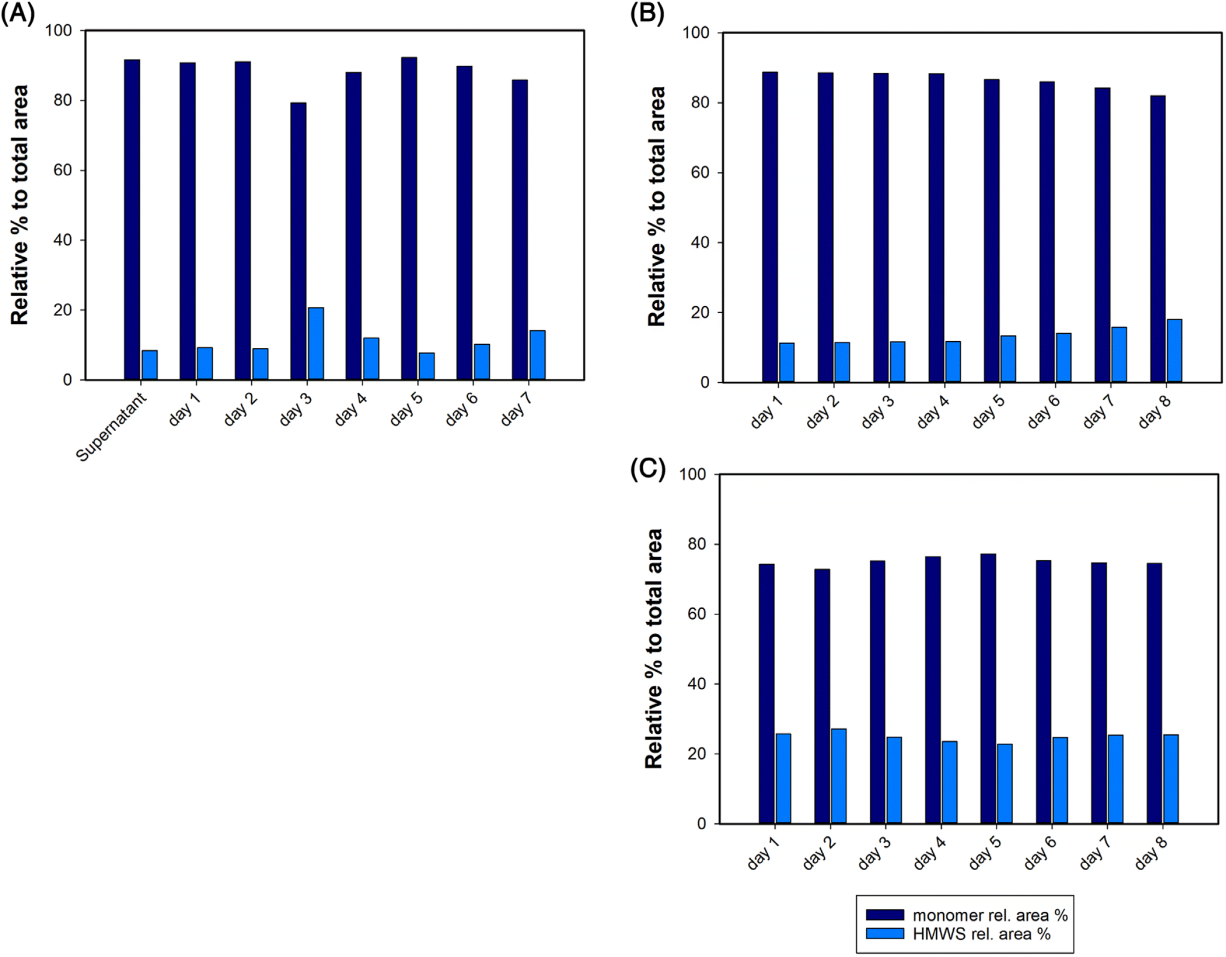
Determination of aggregates and monomer (%) related to total area obtained via analytical size‐exclusion chromatography for cell culture supernatant and re‐dissolved trastuzumab samples obtained during the processes, incubated at low pH for 1 h (viral inactivation). (A) aggregates and monomer (%) for the stand‐alone capture over 7 days of continuous process. (B) aggregates and monomer (%) for the integrated perfusion‐capture over 8 days of process. (C) aggregates and monomer (%) for cell culture supernatant samples from the perfusion process over 8 days of process integration for the integrated perfusion‐capture.

**FIGURE 7:**
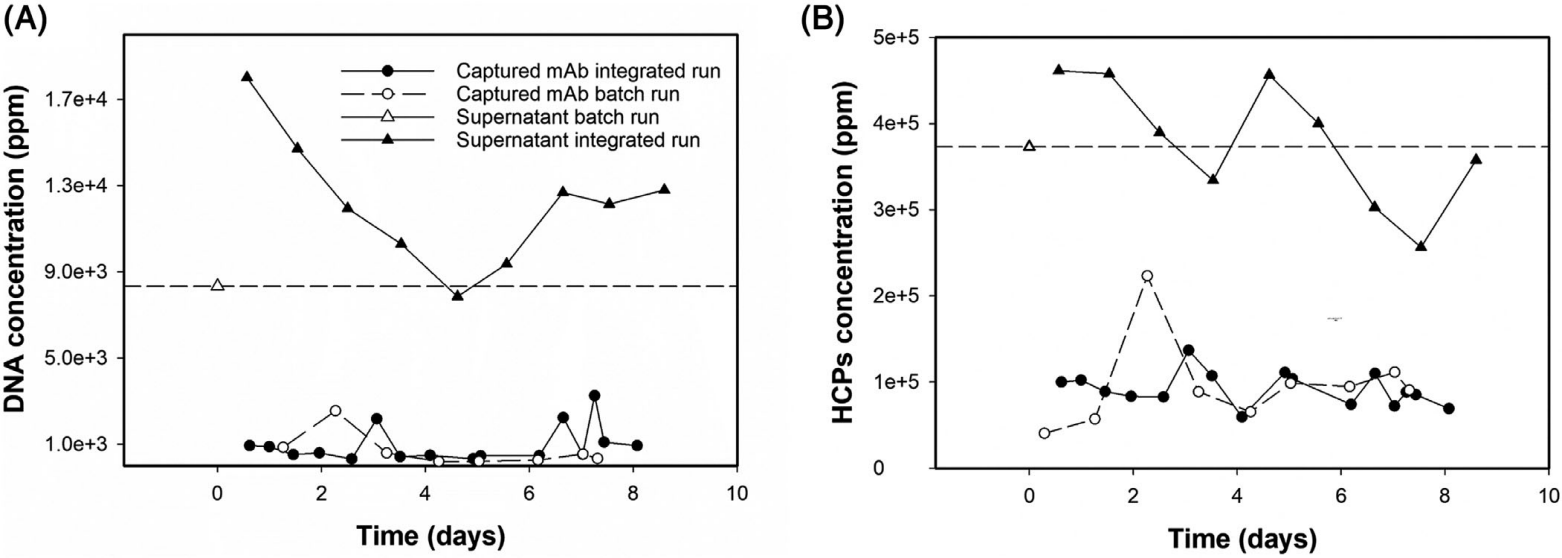
Concentration (ppm) of hcDNA (A) and HCPs (B) for the cell culture supernatant used as feed for the stand‐alone capture (white triangle, dashed line), for the re‐dissolved trastuzumab obtained from the stand‐alone capture (white circles, dashed line), for the cell culture supernatant from the perfusion process integrated with the capture unit (black triangles, solid line), for the re‐dissolved trastuzumab obtained from the integrated perfusion capture (black circles, solid line).

Nonetheless, the resulting product purity was comparable for the two processes, constant over time and between 85% and 95% (Figure 6). For both processes the final concentration of hcDNA and HCPs after capture was in average 30 and 50,000 ppm, respectively. Noteworthy, during the integrated perfusion‐capture process the impurities content in the CCCS varied for all days, while in the stand‐alone process we used a pool and thus a constant composition of the CCCS throughout the process. Even so, we obtained for both processes a similar level of impurities remnants (Figure 7A,B). This highlights the robustness of the precipitation step: the solubility of POI and impurities is determined by the precipitation conditions. For this reason, the ratio of such components after precipitation (when identical precipitation conditions are applied) is expected to be constant, as found in our experiments. The final purity achieved in this study was lower as reported by Sommer et al. [9]. This is explained by the partial co‐precipitation of HCPs and product determined during process development with our cell culture supernatant. Tailoring of cell lines and antibodies for key downstream process parameters is today possible by implementation of high‐throughput screening technologies [36]. Likewise, screening of precipitation conditions can also be performed in a high‐throughput fashion with milli‐fluidic devices, reducing time and material for process development [37]. Thus, separation efficiency of precipitation could be largely improved by tackling the co‐precipitation issue at an early stage. On the other hand, a deeper mechanistical understanding of the precipitation mechanism for different precipitating agents could in future offer new ways to enhance the selectivity of precipitation‐based processes.

### 3.5 RTD of continuous precipitation and TFF

**FIGURE 8:**
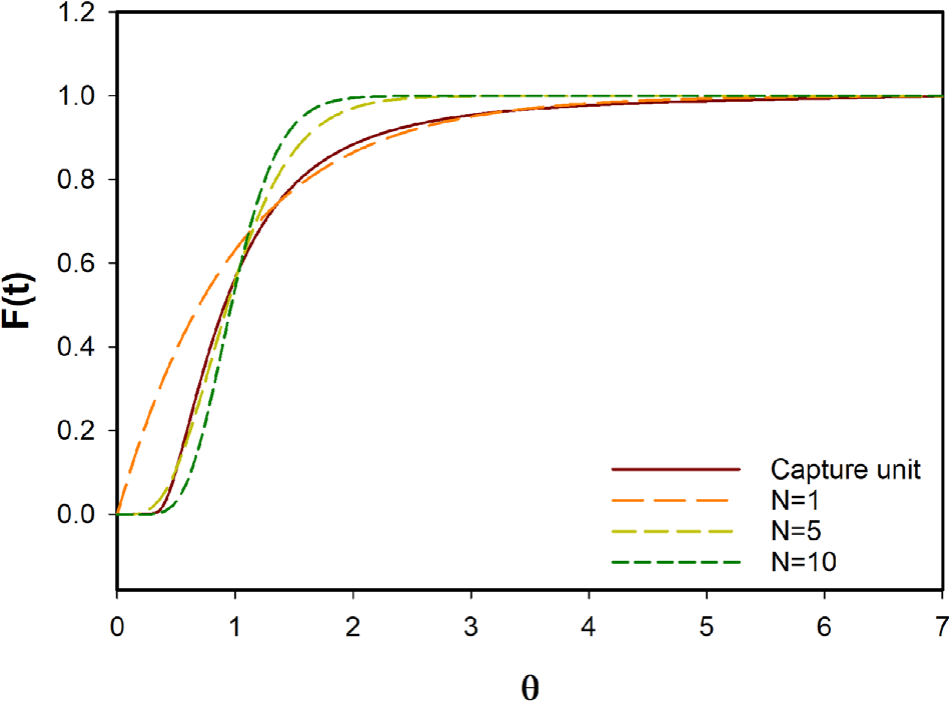
Capture unit cumulative distribution function obtained via salt pulse injection (tubular reactor for antibody precipitation and two stages of TFF. Black solid line, experimental data; orange dashed line, yellow dashed line, black dashed line, theoretical cumulative distribution function obtained by application of the tank‐in‐series model for a series of CSTRs equal to 1, 5, and 10 CSTRs, respectively.

We determined the RTD of our system where the antibody is found in the form of precipitate (the PEG_6000_ tubular reactor and the two stages of TFF) by pulse injection experiments. Previous works already proved that the behavior of the precipitated antibody can be assimilated to the behavior of salt when static mixers are used in tubular reactors [38]. Thus, we used NaCl as tracer for our experiments. Approximately 7 reactor volumes (θ) were needed to flush out all the salt introduced in the system (Figure 8). The mean residence time at the given process flow rates and system volume is equal to 15.4 h. However, this time could be drastically reduced depending on the overall system volume, the upstream scale and the desired conversion rates in the two TFF stages (expressed as percentage of feed to bleed flow rates in each stage). As an example, in our process we applied a conversion rate of 95%. Depending on the level of soluble impurities, a conversion rate of 90% or less could be applied. In this case, the mean residence time would halve to 7.8 h. Furthermore, considering a continuous process of 30 days or more, the reported mean residence time becomes negligible compared to process duration and compared to the mean residence time of the product in the perfusion bioreactor.

### 3.6 Removal of HCPs and hcDNA by flow‐through AEX chromatography

**FIGURE 9:**
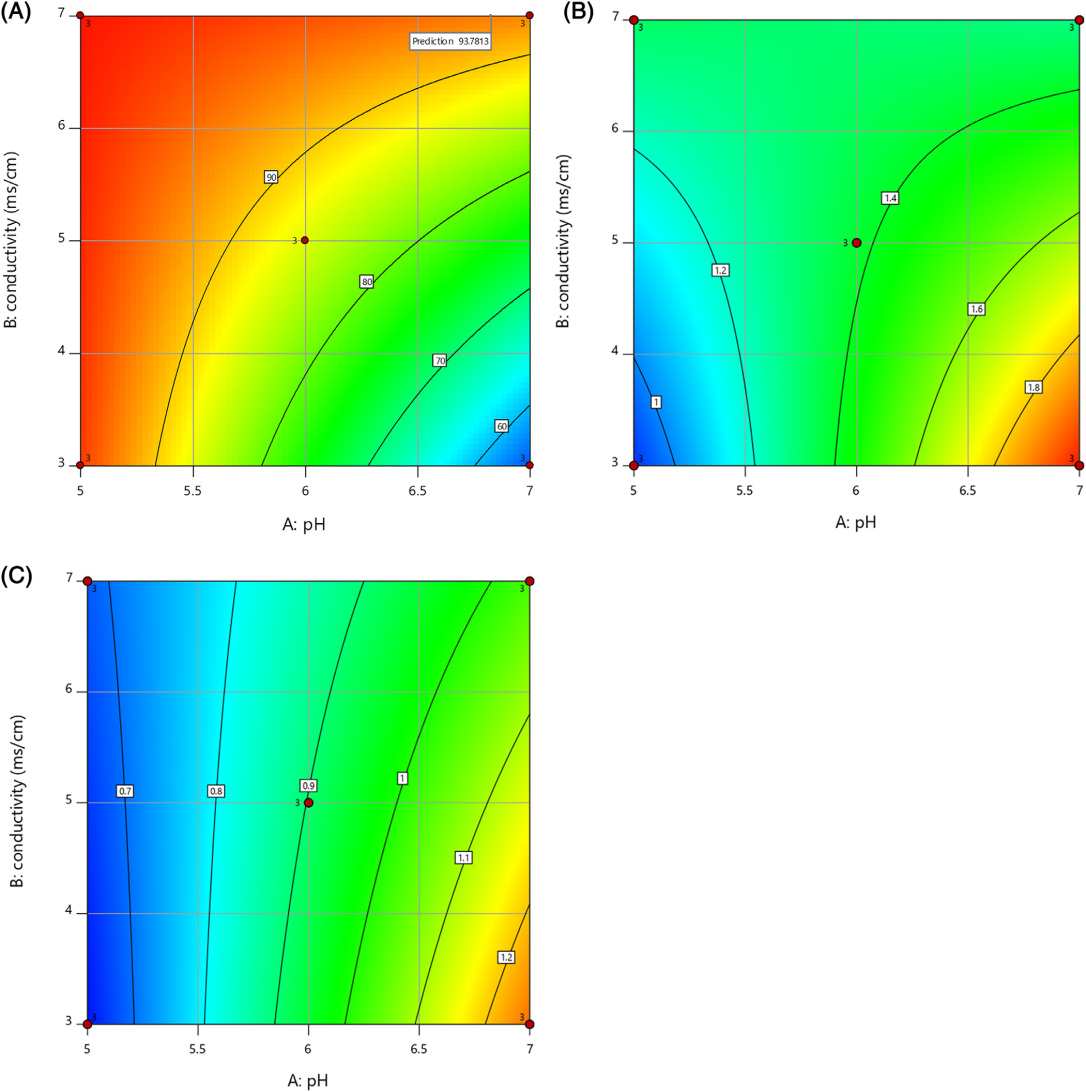
Contour plots of the DoE optimization runs for the flow‐through anion exchange step on re‐dissolved trastuzumab captured during the integrated perfusion‐capture process. (A) step yield (%). (B) logarithmic reduction values (LRVs) for hcDNA. (C) LRVs for HCPs.

The clarified stream after capture and redissolution of trastuzumab produced during the integrated process was further purified in flow through mode with the 3 M Polisher ST. In the best case (pH 7.0, conductivity 3.0 mS/cm), we obtained a logarithmic reduction value (LRV) of 1.3 and 2.0 for HCPs and hcDNA, respectively. However, such LRVs come at the expense of the yield, which was below 60%. One reason for this trade‐off is the extensive dilution required to lower the conductivity to 3 mS/cm. The results from the DoE optimization runs for yield (%) and for HCPs and hcDNA (LRVs) are graphically represented in Figure 9.

The reduction of HCPs and hcDNA given by the integrated capture and a selected set of experiments from the FT‐AEX DoE is summarized in Table 2. The combination of the precipitation‐based capture with FT‐AEX demonstrated a great purification potential. However, similarly to the state‐of‐the‐art platform for monoclonal antibodies, a second polishing step prior to virus filtration and formulation is needed (i.e., HIC or CEX) to lower the impurities level below the threshold limit.

**TABLE 2:** Reduction of HCPs and hcDNA from CCCS to flow‐through AEX for trastuzumab produced in the integrated perfusion‐capture process.

	HCPs (ppm)	HCPs (LRV)	hcDNA (ppm)	hcDNA (LRV)	mAb step yield (%)
CCCS	3.8 · 10^5^	–	8.3 · 10^2^	–	91.4
Capture	3.7 · 10^4^	1	110	1.9	68.7
FT‐AEX	1.8 · 10^2^	1.3	50	0.3	89.9
Total	–	2.3	–	2.2	56.4

### 3.7 Glycosylation analysis

**FIGURE 10:**
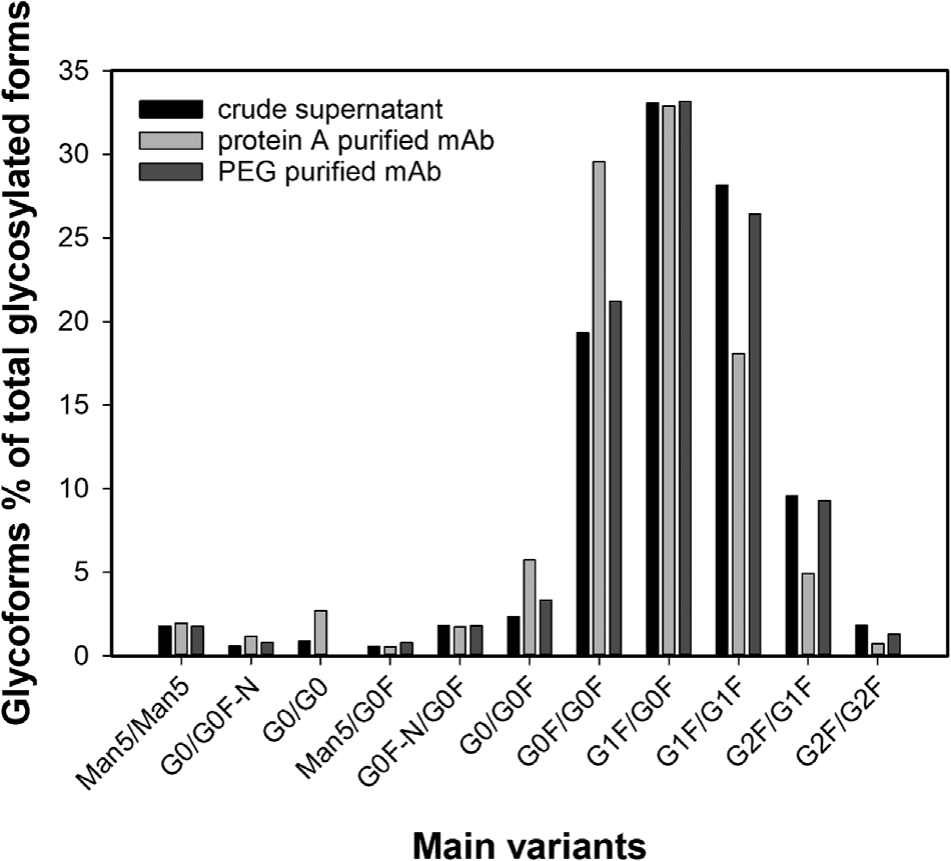
Trastuzumab glycosylation pattern profiles obtained via LC/MS for perfusion cell culture supernatant (black bars), after protein A capture (light grey bars) and after PEG capture and FT‐AEX (dark grey bars).

We assessed and compared the glycosylation pattern of trastuzumab in our CCCS to the glycosylation pattern of the same antibody purified either via preparative protein A affinity chromatography or PEG precipitation (Figure 10). For both purification modalities, we observed a similar glycosylation profile to the CCCS for the main glycoforms variants. High‐mannose glycoforms did not vary between both purification methods. This indicates that no enrichment or depletion of specific glycoforms was promoted by the two purification processes. For complex‐type glycoforms, the protein A purified trastuzumab showed a shift in the relative abundance of G0F/G0F and G1F/G1F. However, a different batch of perfusion cell culture supernatant was used for preparative protein A and no direct comparison can be carried out. As suggested from the FDA guidelines, batch‐to‐batch variability of the glycosylation pattern over repeated production campaigns remains to be assessed.

## 4 CONCLUDING REMARKS

In the present work, we proved the feasibility of using continuous precipitation and filtration techniques for the capture and separation of recombinant antibodies from CCCS over 8 days of process. We obtained a continuous harvest of product, thus an uninterrupted mass flow, utilizing filters in tandem. We directly integrated the downstream train with a perfusion bioreactor in a completely membrane‐based, pool‐less and continuous process for recombinant antibodies. The glycosylation profile of the antibody has not been altered throughout the entire process and process duration. We conclude that it is possible to run an integrated system consisting of a perfusion bioreactor, continuous precipitation and membrane filtration, redissolution and polishing without surge tanks and in an entirely flow‐through mode. This substantially reduces the RTD and enables a fast ramp‐up and shut‐down. In this way, a truly continuous operation can be achieved.

## CONFLICT OF INTEREST STATEMENT

The authors declare no conflicts of interest.

## Data Availability

The data that support the findings of this study are available from the corresponding author, Alois Jungbauer, upon reasonable request.
